# Association of Health Insurance Literacy With Financial Hardship in Patients With Cancer

**DOI:** 10.1001/jamanetworkopen.2022.23141

**Published:** 2022-07-25

**Authors:** Nandita Khera, Nan Zhang, Talal Hilal, Urshila Durani, Minji Lee, Rema Padman, Sandeep Voleti, Rahma M. Warsame, Bijan J. Borah, K. Robin Yabroff, Joan M. Griffin

**Affiliations:** 1 Department of Internal Medicine, Mayo Clinic, Phoenix, Arizona; 2 Division of Hematology/Oncology, University of Mississippi, Jackson; 3 Mayo Clinic, Rochester, Minnesota; 4 Carnegie Mellon University, Pittsburgh, Pennsylvania; 5 American Cancer Society, Kennesaw, Georgia

## Abstract

**Importance:**

Patient-reported financial hardship is an increasing challenge in cancer care delivery. Health insurance literacy and its association with financial hardship in patients with cancer, especially after controlling for financial literacy, have not been well examined.

**Objective:**

To examine the prevalence of and factors in the association between health insurance literacy and financial literacy as well as the overall and individual domains of financial hardship and their association with health insurance literacy, both independently and when adjusted for financial literacy, in patients with cancer.

**Design, Setting, and Participants:**

This cross-sectional survey study recruited and enrolled patients from 2 separate ambulatory infusion centers at Mayo Clinic Arizona in Phoenix, Arizona, and the University of Mississippi Medical Center in Jackson, Mississippi. Adult patients aged 18 years or older were enrolled from December 2019 to February 2020 and from August to October 2020 at Mayo Clinic Arizona (n = 299) and from September 2020 through January 2021 at the University of Mississippi Medical Center (n = 105). Survey respondents received a $5 gift card.

**Exposures:**

Surveys included questions about sociodemographic characteristics, health insurance literacy and financial literacy, financial knowledge, and financial hardship and its domains (material hardship, psychological hardship, and behavioral hardship).

**Main Outcomes and Measures:**

Financial hardship was assessed using the COST–FACIT (Comprehensive Score for Financial Toxicity–Functional Assessment of Chronic Illness Therapy) measure and National Health Interview Survey questions to capture information about the domains of financial hardship. The Health Insurance Literacy Measure is a validated 21-item measure of a consumer's ability to select and use health insurance. Five questions from the National Financial Capability Study assessed financial literacy.

**Results:**

A total of 404 participants were enrolled in the study. Median (IQR) age of the respondents was 63 (54-71) years, and 219 were women (54%), 307 were non-Hispanic White individuals (76%), 153 (38%) had private insurance, and 289 (72%) had solid tumors. Overall financial hardship (denoted by median COST–FACIT score <27 points) was reported by 49% (95% CI, 44%-53%) of the cohort. Prevalence of financial hardship was higher using the National Health Interview Survey questions, with 68% (95% CI, 63%-72%) of respondents reporting at least 1 hardship domain (n = 276). Sixty-six percent (95% CI, 60%-69%) of respondents (n = 265) had a high level of financial literacy. The mean (SD) Health Insurance Literacy Measure score was 64.9 (13.3) points. In multivariable analyses, each 10-point increase in the Health Insurance Literacy Measure score was associated with lower odds of financial hardship (odds ratio, 0.82; 95% CI, 0.68-0.99; *P* = .04). However, this association was no longer significant after adjusting for financial literacy.

**Conclusions and Relevance:**

Results of this study showed that, despite a high level of health insurance literacy and financial literacy, the prevalence of financial hardship was high. Although there were lower odds of financial hardship with increased health insurance literacy, the association was no longer significant when financial literacy was added to the model, suggesting that a high level of financial literacy may help mitigate the adverse outcome of lower health insurance literacy levels in patients with cancer.

## Key Points

**Question:**

Is there an association between health insurance literacy and patient-reported financial hardship independently and after considering financial literacy?

**Findings:**

In this survey study of 404 adult patients with cancer, prevalence of financial hardship ranged from 48% to 68%. There was a modest inverse association between health insurance literacy and financial hardship, which diminished and was no longer statistically significant after controlling for financial literacy.

**Meaning:**

Findings of this study suggest that a high level of financial literacy may help mitigate the adverse outcome of lower health insurance literacy levels in patients with cancer.

## Teaser

This survey study evaluates the association of health insurance literacy and financial literacy with self-reported material, psychological, and behavioral financial hardship among patients with cancer.

## Introduction

Patient-reported financial hardship from cancer treatment is a growing challenge.^1,2,3,4,5,6,7^ Financial hardship is a multidimensional construct that includes material hardship, psychological burden, and adverse coping behaviors related to costs of care.^8^ Several studies have found an association between financial hardship, especially behavioral hardship or avoidance of care, and a lower health insurance literacy level.^9,10,11,12^ Health insurance literacy is defined as “the degree to which individuals have the knowledge, ability, and confidence to find and evaluate information about health plans, select the best plan for their own financial and health circumstances, and use the plan once enrolled.”^13^ To our knowledge, previous studies have not examined the role of financial literacy, a broader concept that includes skills and behaviors for making well-informed financial decisions, in the association between health insurance literacy and financial hardship.^14,15^ Patients’ financial knowledge can help them devise strategies to manage medical expenses amid other financial pressures. There is a paucity of evidence about the prevalence and correlates of health insurance literacy and financial literacy among patients with cancer. In the general population, health insurance and financial literacy are associated; however, it is possible that this association may be more discordant in patients with cancer. Learning by doing may improve health insurance literacy even if overall financial literacy is poor.^16^

**Figure 1.**
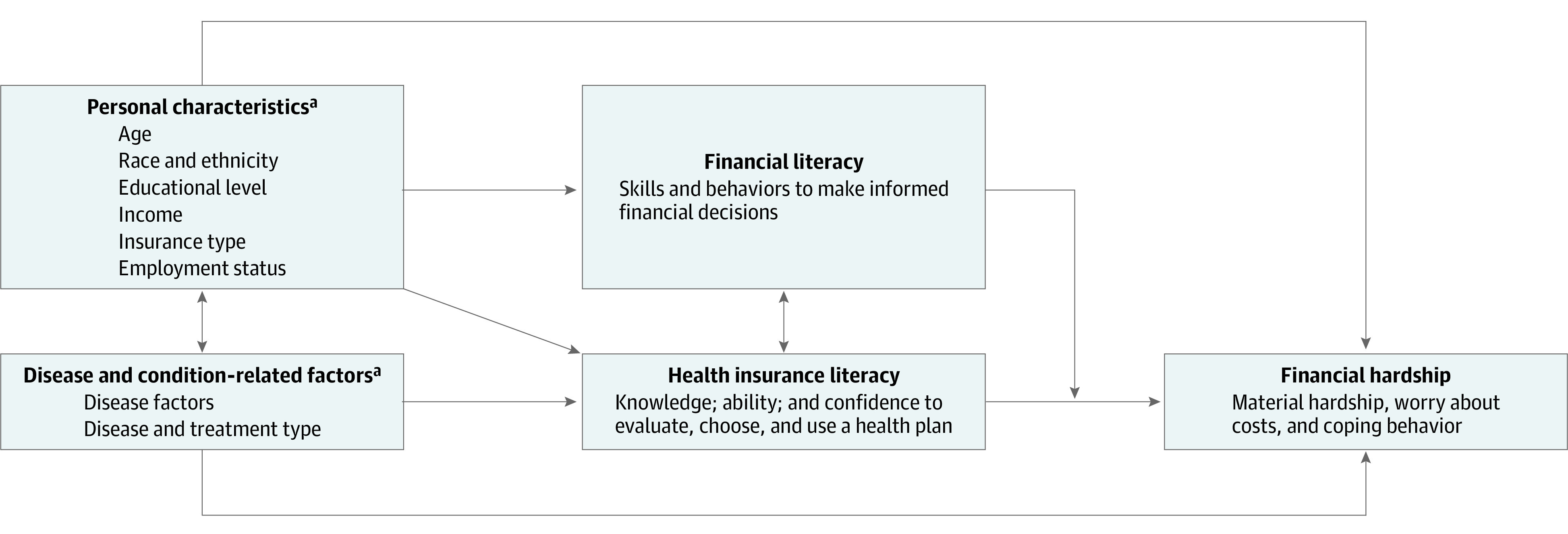
Financial Hardship and Its Proposed Association With Health Insurance Literacy and Financial Literacy ^a^Known risk factor for financial hardship.

The field of financial hardship is now shifting toward interventions.^17,18^ Understanding the association between health insurance literacy and financial hardship and how financial literacy may change this association (Figure 1) is important because these factors are potentially modifiable. In this study, we aimed to examine the prevalence of and factors in the association between health insurance literacy and financial literacy in patients with cancer. We also examined the overall and individual domains of financial hardship and their association with health insurance literacy both independently and when adjusted for financial literacy. We hypothesized that low health insurance literacy was associated with increased financial hardship and that financial literacy may change this association.

## Methods

This survey study was approved by the institutional review boards at the Mayo Clinic Arizona and University of Mississippi Medical Center (UMMC). All participants signed the informed consent form. We followed the American Association for Public Opinion Research (AAPOR) reporting guideline.

All consecutive adult patients (aged >18 years) with cancer coming to the 2 ambulatory infusion centers for treatment were approached and invited to participate in the study. At Mayo Clinic Arizona in Phoenix, Arizona, approximately 65% of patients were from the state and 35% were from out of state or countries outside the US, whereas most patients at UMMC in Jackson, Mississippi, were local. Patients were enrolled from December 2019 to February 2020 and from August to October 2020 at Mayo Clinic Arizona (only local or regional patients) and from September 2020 through January 2021 at UMMC. Participants who completed the study surveys (eAppendix in the Supplement) were provided a $5 gift card. Response rate was calculated using AAPOR response rate formula RR2.^19^

### Study Variables

Age, sex, and insurance type were collected from the electronic medical record at each site. Additional sociodemographic characteristics, including household size, race and ethnicity (including non-Hispanic White and other categories, which included a small number of respondents), educational level, marital status, employment status, and monthly household income, were self-reported in and collected from the study surveys. Disease data, such as solid tumor vs hematologic malignant neoplasm, time from diagnosis, lines of treatment (including chemotherapy, surgery, and radiotherapy), and receipt of specific treatments, were collected from the electronic medical record.

### Study Outcomes

Self-reported financial hardship was assessed using the COST–FACIT (Comprehensive Score for Financial Toxicity–Functional Assessment of Chronic Illness Therapy), a validated 12-item measure.^20^ The total COST–FACIT score can range from 0 to 44 points, with a lower score indicating greater financial hardship. In the first survey, the median COST–FACIT score (derived from the study sample) was used to stratify the presence or absence of overall financial hardship (lower than median scores indicated hardship) as recommended.^7,21^ Questions from the National Health Interview Survey (NHIS) helped capture information about all 3 hardship domains.^22^ Material hardship was measured as difficulty with paying medical bills, psychological hardship as worry about costs of health care, and behavioral hardship as delayed and/or forgoing care because of worry about costs. A dichotomous summary measure for each hardship domain indicated the presence or absence of financial hardship.

The Health Insurance Literacy Measure (HILM) is a validated 21-item measure of a consumer's ability to select and use health insurance.^13^ Scores can range from 21 to 84 points, with higher scores indicating greater levels of health insurance literacy. In the second survey, a HILM score lower than 60 points indicated a low level of health insurance literacy for descriptive analyses, and continuous scores were used for the multivariable analyses, similar to a previous report.^9^

Five questions from the National Financial Capability Study covering aspects of economics and finance encountered in everyday life were used to assess financial literacy.^23^ These concepts include compound interest, inflation, principles of risk and diversification, the relationship between bond prices and interest rates, and payments over the life of a mortgage. Correct answers to more than 3 of the 5 questions in the third survey indicated a high level of financial literacy. Self-reported financial knowledge was assessed on a scale of 1 (very low) to 7 (very high).

### Statistical Analysis

At Mayo Clinic Arizona, differences between study participants and nonparticipants were compared using analysis of variance and Pearson χ^2^ test. Information about the nonrespondents was not available for the UMMC cohort because collecting these data was not allowed by the UMMC institutional review board. We used χ^2^ tests and unpaired, 2-tailed *t* tests to compare the sociodemographic and clinical characteristics of respondents with financial hardship vs those without financial hardship. A *P* = .05 was chosen as the cutoff criterion for statistical significance, and all tests were 2-sided.

Pearson correlation coefficient was used to describe the association between the COST–FACIT score and financial hardship domains from the NHIS questions. Spearman correlation coefficients among the financial literacy score, self-reported financial knowledge, and overall HILM scores were calculated.

The backward model selection method using Akaike information criteria was used to select important variables to build a multivariable logistic regression model for health insurance literacy and financial hardship. We used the COST–FACIT score as the primary outcome for the model because it is a validated instrument. This model assessed the association of health insurance literacy (using HILM score as a continuous variable: every 10-point increase) with financial hardship (median COST–FACIT score <27 points) while adjusting for age, sex, time from diagnosis, race and ethnicity, household size, educational level, and monthly household income because these factors have been shown to be associated with the occurrence of financial hardship. In sequential analyses, we added the financial literacy score to the model.

In a sensitivity analysis, alternative models were examined that used a different COST–FACIT threshold score of 21 (the threshold used in the study by De Souza et al^21^) and the COST–FACIT score as a continuous variable. Parallel logistic regression models were constructed to investigate the association between HILM score and NHIS measures in a secondary analysis. A stratified analysis by the 2 sites was performed because of the differences in distribution of their sociodemographic variables. All analyses were conducted with Rstudio, version 4.0.3 (RStudio, PBC).

## Results

A total of 299 patients were enrolled of the 352 patients who were approached at Mayo Clinic Arizona (85% response rate), and 105 patients were enrolled of the 167 invited at UMMC (63% response rate). Overall, there were 404 participants. There were no significant differences in age, sex, race and ethnicity, and cancer type between nonrespondents (n = 53) and respondents (n = 299) at Mayo Clinic Arizona. Respondents had a median (IQR) age of 63 (54-71) years, and the sample consisted of 219 women (54%) and 185 men (46%), of whom 307 (76%) were non-Hispanic White individuals, 208 (51%) reported having a college degree or higher educational level, 94 (23%) were working full-time or part-time, and 153 (38%) had private insurance. Seventy-two percent of respondents (n = 289) had solid tumors, 110 (42%) of whom had metastatic disease (Table 1).

**Table 1.** Differences in Clinical and Sociodemographic Characteristics of Respondents Without vs With Financial Hardship, Based on the Comprehensive Score for Financial Toxicity–Functional Assessment of Chronic Illness Therapy Measure

Variable	No. (%)			*P* value
	Without financial hardship (n = 208)	With financial hardship (n = 196)	Total (N = 404)	
Age at enrollment, mean (SD), y	66 (12.2)	57 (12.9)	62 (13.4)	<.001^a^
Race and ethnicity^b^				
Non-Hispanic White	180 (87)	127 (65)	307 (76)	<.001^d^
Other^c^	27 (13)	69 (35)	96 (24)	
Sex				
Male	109 (52)	76 (39)	185 (46)	.006^d^
Female	99 (48)	120 (61)	219 (54)	
Monthly household income, $				
<4999	66 (32)	132 (68)	198 (50)	<.001^d^
≥5000	140 (68)	61 (32)	201 (50)	
Household size, mean (SD)	2.2 (0.9)	2.6 (1.5)	2.4 (1.3)	.009^e^
Educational level				
≥Any college	132 (64)	76 (39)	208 (51)	<.001^d^
<College	76 (36)	120 (61)	196 (49)	
Employment status				
Unemployed	1 (1)	2 (1)	3 (1)	<.001^d^
Homemaker, retired, or student				
Age <65 y	36 (17)	57 (30)	93 (23)	
Age ≥65 y	113 (54)	50 (25)	163 (40)	
On leave of absence, but still employed	14 (7)	37 (19)	51 (13)	
Working full-time or part-time	44 (21)	50 (25)	94 (23)	
Marital status				
Married	159 (76)	109 (56)	268 (66)	<.001^d^
Widowed, divorced, separated, or never married	49 (24)	87 (44)	136 (34)	
Insurance type				
None	0 (0)	6 (3)	NA	.001^d^
Public				
Medicare, with and without secondary insurance	129 (62)	69 (35)	198 (49)	
Medicaid, state-based program	15 (7)	28 (14)	43 (11)	
VA, TRICARE	1 (1)	2 (1)	3 (1)	
Private	63 (30)	90 (46)	153 (38)	
Cancer type				
Solid tumor	143 (69)	146 (74)	289 (72)	.20^d^
Hematologic cancer	65 (31)	50 (26)	115 (28)	
Disease status for solid tumor				
Nonmetastatic	73 (57)	78 (59)	151 (58)	.27^d^
Metastatic	56 (43)	54 (41)	110 (42)	
Time from diagnosis, y				
<1	76 (37)	100 (51)	176 (44)	<.001^d^
1-5	76 (36)	71 (36)	147 (36)	
>5	56 (27)	25 (13)	81 (20)	
Total lines of treatment, mean (SD)	3.9 (2.7)	3.2 (2.1)	3.6 (2.4)	.02^e^
Site location				
Mayo Clinic Arizona	175 (84)	124 (63)	299 (74)	<.001^d^
UMMC	33 (16)	72 (37)	105 (26)	
Survey period				
Before COVID-19 pandemic	114 (55)	86 (44)	200 (50)	.03^f^
During COVID-19 pandemic	94 (45)	110 (56)	204 (50)	

Abbreviations: NA, not applicable; UMMC, University of Mississippi Medical Center; VA, US Department of Veterans Affairs.

^a^ Calculated with linear model analysis of variance.

^b^ Race and ethnicity were self-reported in the surveys.

^c^ Other race and ethnicity categories were compared with the non-Hispanic White category because of the small number of respondents in the other categories.

^d^ Calculated with Pearson χ^2^ test.

^e^ Calculated with Kruskal-Wallis rank sum test.

^f^ Calculated with Fisher exact test for count data.

### Prevalence of Financial Hardship

**Figure 2.**
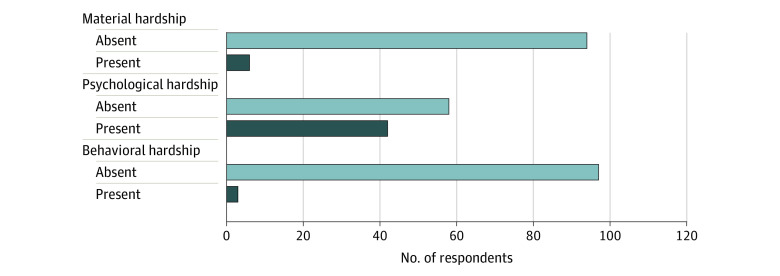
Distribution of Psychological, Behavioral, and Material Hardship Domains in Respondents Without Financial Hardship

Overall, 49% (n = 196 of 404; 95% CI, 44%-53%) of the study cohort had a median COST-FACIT score of 27 points (financial hardship was denoted by a score of <27 points). Prevalence of financial hardship was higher using the NHIS questions: 68% (n = 276; 95% CI, 63%-72%) of respondents reported at least 1 hardship domain. Material hardship was reported by 28% (n = 6), psychological hardship by 64% (n = 42), and behavioral hardship by 13% (n = 3) of the overall cohort. Figure 2 shows the distribution of material, psychological, and behavioral hardship domains for patients without financial hardship using the COST-FACIT measure. Approximately 40% of respondents without financial hardship endorsed psychological hardship based on the NHIS question.

Table 1 shows the distribution of sociodemographic and clinical characteristics among respondents with and without financial hardship. Younger age, being a patient from UMMC, female sex, other race and ethnicity, cancer diagnosis, higher number of household members, lower educational level, nonmarried status, and lower monthly household income were significantly associated with financial hardship. There was no difference in financial hardship based on type of cancer (solid tumor vs hematologic cancer), metastatic vs nonmetastatic disease, or type of treatment.

### Financial Literacy and Its Association With Financial Hardship

The median (IQR) score of the overall cohort for financial knowledge was 5.0 (4.0-6.0) points. Sixty-six percent (95% CI, 60%-69%) of respondents (n = 265) correctly answered more than or equal to 4 financial literacy questions, indicating high financial literacy. Factors associated with low financial literacy included being a patient from UMMC (odds ratio [OR], 3.04; 95% CI, 1.67-5.55; *P* < .001); female sex (OR, 2.02; 95% CI, 1.21-3.41; *P* = .008); other race and ethnicity (OR, 2.10; 95% CI, 1.13-3.90; *P* = .02); educational level less than a college degree (OR, 3.44; 95% CI, 2.03-5.91; *P* < .001); and monthly household income of less than $5000 (OR, 2.00; 95% CI, 1.17-3.44; *P* = .01).

Self-reported financial knowledge had a significant medium correlation with financial literacy (Pearson *r* = 0.36; *P* < .001). Patients with financial hardship had a lower median score for self-assessed financial knowledge (5.0 vs 6.0 points; *P* < .001) compared with those without financial hardship. Financial hardship was reported by a higher proportion of respondents with low financial literacy (49% of those with financial hardship [n = 94] vs 23% of those without financial hardship [n = 45]; *P* < .001). Self-assessed financial knowledge and financial literacy scores were lower in respondents with financial hardship vs those without financial hardship (eFigure in the Supplement).

### Health Insurance and Financial Literacy and Their Association With Financial Hardship

The mean (SD) HILM score was 64.9 (13.3) points. Patients from other race and ethnicity categories had higher odds of having a low health insurance literacy level (OR, 2.11; 95% CI, 1.26-3.53; *P* = .004). Although time from diagnosis was not associated, patients with more lines of treatment had lower odds for a low level of health insurance literacy (OR, 0.8; 95% CI, 0.7-0.9; *P* = .007). The correlation between health insurance literacy and financial literacy was medium although statistically significant (Pearson *r* = 0.28; *P* < .001). Both health insurance literacy and financial literacy were low in 15% of all respondents (n = 61) and high in 50% of respondents (n = 202), whereas 14% had a high level of financial literacy but a low level of health insurance literacy (n = 57), and 21% had a high level of health insurance literacy but a low level of financial literacy (n = 85).

**Figure 3.**
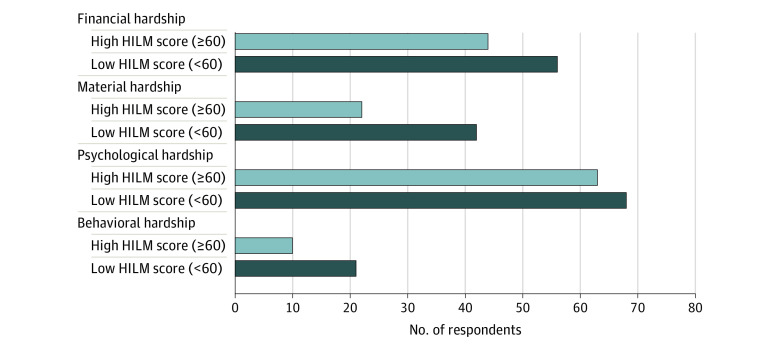
Association of Health Insurance Literacy With Financial Hardship and Material, Psychological, and Behavioral Hardship Domains Financial hardship was assessed using the COST–FACIT (Comprehensive Score for Financial Toxicity–Functional Assessment of Chronic Illness Therapy) measure. Material, psychological, and behavioral hardship domains were based on National Health Interview Survey questions. HILM indicates Health Insurance Literacy Measure.

Respondents with financial hardship had a higher mean (SD) HILM score of 62.7 (13.1) points compared with a mean (SD) score of 67.0 (13.2) points in those no financial hardship (*P* < .001). A lower health insurance literacy level (HILM score <60 points) in contrast to a higher level (HILM score >60 points) was associated with overall financial hardship (58% vs 44%; *P* = .02) as well as material (42% vs 22%; *P* < .001) and behavioral (21% vs 10%; *P* = .005) hardship but not psychological hardship (68% vs 63%; *P* = .28) domains (Figure 3).

In multivariable analyses, each 10-point increase in HILM score was associated with lower odds of financial hardship (OR, 0.82; 95% CI, 0.68-0.99; *P* = .04) (Table 2). However, this association was no longer significant after adjusting for financial literacy. Similar results were obtained with the models that used alternative COST–FACIT score thresholds (eTable 1 in the Supplement).

**Table 2.** Association of Health Insurance Literacy With Financial Hardship by the Comprehensive Score for Financial Toxicity–Functional Assessment of Chronic Illness Therapy Measure

Variable^**b**^	Model 1		Model 2^**a**^	
	OR (95% CI)	*P* value	OR (95% CI)	*P* value
HILM overall score (10-point increase)	0.82 (0.68-0.99)	.04	0.88 (0.72-1.09)	.24
Financial literacy score (1-point increase)	NA	NA	0.86 (0.69-1.07)	.17

Abbreviations: HILM, Health Insurance Literacy Measure; NA, not applicable; OR, odds ratio.

^a^ Model 2 included financial literacy.

^b^ Other significant variables included patient age, time from diagnosis, educational level, and income in model 1 and patient age, time from diagnosis, and income in model 2.

A model that used NHIS domains as the outcome showed similar findings: for every 10-point increase in the HILM score, the odds of affecting any (vs 0) financial hardship domains decreased significantly (OR, 0.68; 95% CI, 0.54-0.85; *P* < .001). The association remained significant when financial literacy was added to the model (OR, 0.72; 95% CI, 0.57-0.91; *P* = .005) (eTable 2 in the Supplement).

In a stratified analysis that included only patients from Mayo Clinic Arizona, the results were similar, with a significant association between health insurance literacy and financial hardship (OR, 0.77; 95% CI, 0.6-0.98; *P* = .03). As in the overall cohort, the association between health insurance literacy and financial hardship was no longer significant when financial literacy was added to the model. A stratified analysis using only patients from UMMC did not show an association between health insurance literacy and financial hardship, likely because of lower power owing to a smaller sample size.

## Discussion

Financial hardship from treatment is an enormous issue for patients with cancer that has been associated with treatment nonadherence and higher bankruptcy risk.^24,25^ In this patient cohort, despite reasonable rates of health insurance literacy and financial literacy, the prevalence of financial hardship was high and was associated mainly with psychological hardship. A low level of health insurance literacy has been found to be associated with financial hardship, although there is scant information that financial literacy may alter this association.^9,11,12^ The present study adds to this evidence by highlighting that the material and behavioral (not the psychological) hardship domains are the ones affected by health insurance literacy. We also found that health insurance literacy no longer had a significant association with financial hardship when financial literacy was included in the primary model. This finding suggests that financial literacy with its association with good financial decision-making and increased financial capability for managing overall finances may help in mitigating the adverse implications of a low level of health insurance literacy for financial hardship. The secondary analysis, which used the NHIS-derived measure of financial hardship, found that health insurance literacy was significantly associated with financial hardship even when financial literacy was included in the model. The reason for this finding may be that the overall prevalence of financial hardship was much higher with the NHIS questions. These measures were complementary, reflected different aspects of financial hardship, and were associated with a different outcome, although the implications were similar.

Health insurance literacy may be more relevant to patients who have a need for health care services. In a previous qualitative study, the complexity of insurance coverage and difficulty navigating various options and requirements emerged as factors in financial distress.^26^ In the general population, people with lower educational levels, younger age, and without insurance were more likely to have a low level of health insurance literacy.^27^ We also found an association between health insurance literacy and the number of lines of treatment, suggesting that patients may learn to navigate health care better as they continue their treatment.

Insurance coverage alone is not a guarantee of affordable care. High deductible plans are associated with greater financial hardship for patients with low income.^28^ Health insurance literacy is important for patients when selecting a health plan; making financially sound choices, such as choosing in-network clinicians and low-deductible plans and paying premiums on time to ensure continued coverage; and when using the plan for recommended health services.^9,29^ Patients cannot control the complexity of insurance benefit design or the cost-sharing requirements, which is in the domain of policy makers. However, efforts by health plans, hospitals, and the federal government to improve health insurance literacy can ensure that patients make financially sound choices among different insurance products based on their financial condition and medical requirements, use their benefits optimally, and continue their affordable coverage for recommended cancer care. Financial navigation programs offer a proactive approach to alleviating financial hardship, especially for patients with a low level of health insurance literacy. Culturally appropriate interventions and navigators who are familiar with targeted communities can help address the individual-level barriers.^26,30,31^

A cancer diagnosis is an overwhelming life event, and financial aspects of care add an extra layer of stress. Financial knowledge and the skills to apply that knowledge can help in making sound financial decisions in health or sickness. The National Financial Capability Study showed that, in the general population, only approximately 34% of the respondents were able to answer at least 4 of 5 financial literacy questions compared with 64% in the present cohort.^23^ The results of the present study highlight the need for work upstream of a cancer or other chronic medical diagnosis. Schools, colleges, workplaces, not-for-profit organizations, and governmental agencies need to develop financial education programs to improve financial literacy and foster financial capability, especially in the face of a catastrophic event, such as a cancer diagnosis.

Understanding the modifiable factors associated with financial hardship strengthens the rationale for interventions to improve both health insurance literacy and financial literacy, such as targeted education about insurance terminology, financial options, and financial navigation to facilitate problem-focused coping.^26,30,31^ Results of this study suggest that material or behavioral hardship needs to be an independent outcome of interest, in addition to overall financial hardship, when studying interventions for health insurance literacy and financial literacy.

Consistent with other research, this study showed high rates of financial hardship in patients with cancer.^8^ Psychological hardship was the most common type, which was prevalent in approximately two-thirds of the cohort, as found in previous reports.^22^ Although the COST–FACIT survey did not include a behavioral response question (eg, delaying or forgoing care because of cost), it was able to capture behavioral hardship well, but 40% of participants who reported psychological hardship on the NHIS questions did not have financial hardship based on the COST–FACIT measure. Both overall financial hardship and each hardship domain were higher in the UMMC cohort than in the Mayo Clinic Arizona cohort, which suggests a socioeconomically disadvantaged population. The lower response rate among the UMMC cohort highlighted the challenges of recruiting such patients for a study on this topic.

### Limitations

This study has several limitations. A cross-sectional design, relatively small sample size, and self-reported data potentially introduced recall and reporting biases. The convenience sampling approach may have led to the selection of a specific subset of patients, although we tried to minimize this possibility by inviting consecutive patients at 2 ambulatory infusion centers, including 1 cohort with diverse sociodemographic characteristics, such as insurance type and monthly household income. The lower response rate increased the possibility of nonresponse bias, thereby changing the results of the stratified analysis. It is likely that patients with higher health insurance literacy and financial literacy as well as lower financial hardship may be more motivated to answer the surveys. Information about the nonrespondents was not available for the UMMC cohort. Patients from UMMC were also enrolled during the COVID-19 pandemic, which may have affected their self-reporting of financial hardship. To address this issue, we enrolled an additional 100 patients from Mayo Clinic Arizona in a similar time frame and reported on financial hardship before and during the COVID-19 pandemic. Despite recruitment at 2 cancer centers, patients in the racial and ethnic minority groups made up only one-quarter of the overall cohort. This composition may limit the generalizability of the results to the US population, which is becoming increasingly racially and ethnically diverse.

## Conclusions

In this survey study, increased health insurance literacy was associated with lower odds of financial hardship especially affecting the material and behavioral domains. We also found that high financial literacy may help to mitigate the adverse outcomes associated with lower health insurance literacy.. The findings highlight the need for tailored interventions to help alleviate financial hardship in diverse populations.

## Notes

Supplement. **eFigure.** Association of Financial Knowledge and Financial Literacy With Financial Hardship **eTable 1.** Association of Health Insurance Literacy With Financial Hardship Assessed by COST-FACIT Score as a Dichotomous Variable (Cutoff for Financial Hardship Being <21) and as a Continuous Variable **eTable 2.** Association of Health Insurance Literacy With Financial Hardship Measured by National Health Interview Survey Questions **eAppendix.** Surveys Used in the Study
