# Supplementary material for: Association of Health Insurance Literacy With Financial Hardship in Patients With Cancer
Source: JAMA Netw Open. 2022 Jul 25;5(7):e2223141. doi: 10.1001/jamanetworkopen.2022.23141 (PMC9315419; doi:10.1001/jamanetworkopen.2022.23141)
Supplement: Supplement. — eFigure. Association of Financial Knowledge and Financial Literacy With Financial Hardship eTable 1. Association of Health Insurance Literacy With Financial Hardship Assessed by COST-FACIT Score as a Dichotomous Variable (Cutoff for Financial Hardship Being <21) and as a Continuous Variable eTable 2. Association of Health Insurance Literacy With Financial Hardship Measured by National Health Interview Survey Questions eAppendix. Surveys Used in the Study [file jamanetwopen-e2223141-s001.pdf]

Supplemental Online Content

Khera N, Zhang N, Hilal T, et al. Association of health insurance literacy with financial hardship in patients with cancer. *JAMA Netw Open*. 2022;5(7):e2223141. doi:10.1001/jamanetworkopen.2022.23141

eFigure. Association of Financial Knowledge and Financial Literacy With Financial Hardship

eTable 1. Association of Health Insurance Literacy With Financial Hardship Assessed by COST-FACIT Score as a Dichotomous Variable (Cutoff for Financial Hardship Being <21) and as a Continuous Variable

eTable 2. Association of Health Insurance Literacy With Financial Hardship Measured by National Health Interview Survey Questions

eAppendix. Surveys Used in the Study

This supplemental material has been provided by the authors to give readers additional information about their work.

© 2022 Khera N et al. *JAMA Network Open.*

eFigure. Association of Financial Knowledge and Financial Literacy With Financial Hardship

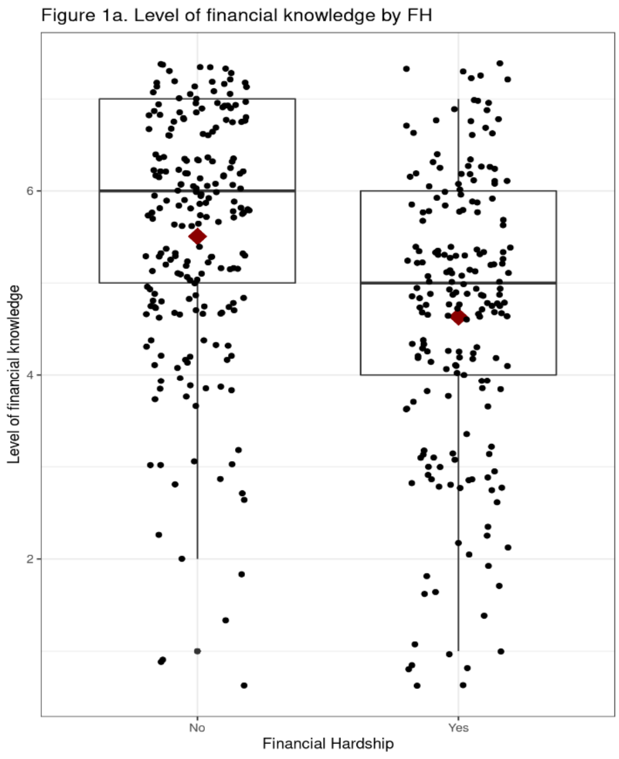

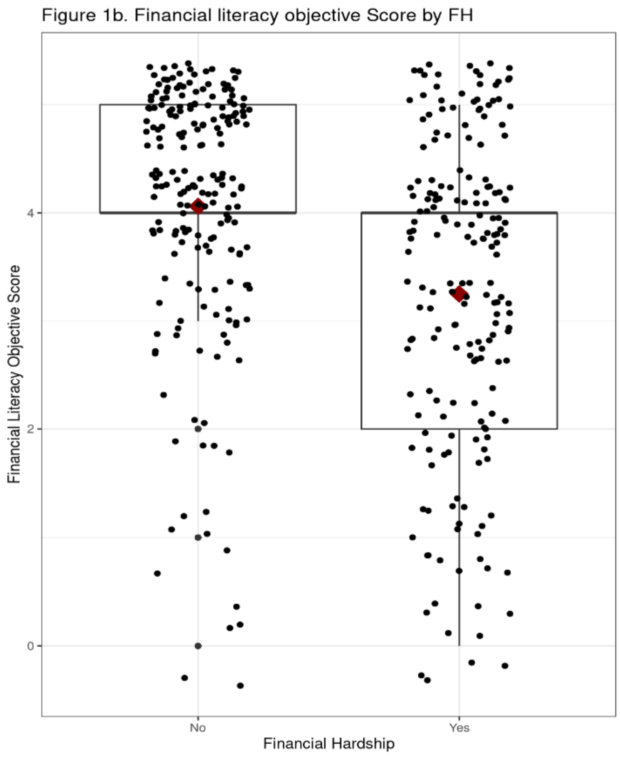

© 2022 Khera N et al. *JAMA Network Open.*

eTable 1. Association of Health Insurance Literacy With Financial Hardship Assessed by COST-FACIT Score as a Dichotomous Variable (Cutoff for Financial Hardship Being <21) and as a Continuous Variable

**Variables**	**Model 1**		**Model 2***	
COST-FACIT score as a dichotomous variable (cutoff for financial hardship <21 median score)				
	**OR (95% CI)**	**p value**	**OR (95% CI)**	**p value**
HILM Overall Score (ten units increase)	0.8 (0.66 - 0.96)	0.018	0.88 (0.71 - 1.08)	0.21
Financial literacy score (one-unit increase)			0.79 (0.63 – 0.97)	0.03
COST FACIT as continuous variable				
	**Beta (95% CI)**	**p value**	**Beta (95% CI)**	**p value**
HILM Overall Score (ten units increase)	1.1 (0.33 – 1.8)	0.005	0.71 (-0.08 - 1.5)	0.08
Financial literacy score (one-unit increase)			0.92 (0.07– 1.8)	0.034

Other significant variables in these models include age, gender, time since diagnosis and income

© 2022 Khera N et al. *JAMA Network Open.*

eTable 2. Association of Health Insurance Literacy With Financial Hardship Measured by National Health Interview Survey Questions

Variables	OR (95% CI)	p value	OR (95% CI)	p value
HILM Overall Score (ten units increase)	0.68 (0.54 - 0.85)	<0.001	0.72 (0.57 - 0.91)	0.005
Financial literacy score			0.87 (0.68-1.11)	0.27

Other significant variables in these models include age, gender, time since diagnosis, household size and income

© 2022 Khera N et al. *JAMA Network Open.*

eAppendix. Surveys Used in the Study

COST-FACIT

Below is a list of statements that other people with your illness have said are important. **Please circle or mark one number per line to indicate your response as it applies to the past 7 days.**

		Not at all	A little bit	Some -what	Quite a bit	Very much
FT1	I know that I have enough money in savings, retirement, or assets to cover the costs of my treatment	0	1	2	3	4
FT2	My out-of-pocket medical expenses are more than I thought they would be	0	1	2	3	4
FT3	I worry about the financial problems I will have in the future as a result of my illness or treatment	0	1	2	3	4
FT4	I feel I have no choice about the amount of money I spend on care	0	1	2	3	4
FT5	I am frustrated that I cannot work or contribute as much as I usually do	0	1	2	3	4
FT6	I am satisfied with my current financial situation	0	1	2	3	4
FT7	I am able to meet my monthly expenses	0	1	2	3	4
FT8	I feel financially stressed	0	1	2	3	4
FT9	I am concerned about keeping my job and income, including work at home	0	1	2	3	4
FT10	My cancer or treatment has reduced my satisfaction with my present financial situation	0	1	2	3	4
FT11	I feel in control of my financial situation	0	1	2	3	4
FT12	My illness has been a financial hardship to my family and me	0	1	2	3	4

© 2022 Khera N et al. *JAMA Network Open.*

1. In the past 12 months did you or anyone in the family have problems paying or were unable to pay any medical bills?
    a. Yes
    b. No
    c. Refused
    d. Don't Know

2. Do you or does anyone in your family currently have any medical bills that are being paid off over time?
    a. Yes
    b. No
    c. Refused
    d. Don't Know

3. How worried are you right now about paying medical costs for your healthcare?
    a. very worried
    b. somewhat worried
    c. not at all worried
    d. Refused
    e. Don't Know

4. During the past 12 months, has your medical care BEEN DELAYED because of worry about the cost?
    a. Yes
    b. No
    c. Refused
    d. Don't Know

5. During the past 12 months, was there any time when you needed medical care, but DID NOT GET IT because you couldn't afford it?
    a. Yes
    b. No
    c. Refused
    d. Don't Know

© 2022 Khera N et al. *JAMA Network Open.*

Health Insurance Literacy Measures©
(HILM)

I. The HILM: Self-Reported Behavior Scales

Scale 1. Behavior: Choosing a Health Plan

The next set of questions are about **comparing health insurance plans**. When answering the questions, please imagine that you have a choice of health plans.

When comparing health insurance plans, how likely are you to…	Not at all likely	Somewhat likely	Moderately likely	Very likely
1. Find out if you have to meet a deductible for health care services? *A deductible is the amount of money you have to pay before your health insurance will pay anything for your health care.*				
2. Look to see which doctors and hospitals are covered in each plan?				
3. Understand what you have to pay for prescription drugs?				
4. Understand what you would have to pay for emergency department visits?				
5. Understand what you would have to pay for specialist visits?				
6. Find out if the plans cover unexpected costs such as hospital stays?				

Scale 2. Behavior: Using a Health Plan

The next questions are about your experiences **using your health insurance plan**, for example, going to the doctor to receive care covered by the health plan.

When using your health insurance plan, how likely are you to …	Not at all likely	Somewhat likely	Moderately likely	Very likely
1. Look into what your health plan will and will not cover before you get health care services?				
2. Look to member services to tell you what medical services your health plan covers?				
3. Find out if a doctor is in-network before you see him/her? *A network is a group of doctors, hospitals, and other health care professionals who have a contract with a health insurance plan to take care of the plan's members.*				

© 2022 Khera N et al. *JAMA Network Open.*

When using your health insurance plan, how likely are you to …	Not at all likely	Somewhat likely	Moderately likely	Very likely
4. Review the statements you get from your health plan showing what you owe and what they paid for a service?				

II. HILM: Self-Confidence Scales

Scale 3. Confidence: Choosing a Health Plan

The next questions are about how confident you feel **choosing a health insurance plan**.

How confident are you that…	Not all confident	Slightly confident	Moderately confident	Very confident
1. You understand health insurance terms?				
2. You know where to find the information you need to choose a health plan if you were not offered insurance through an employer?				
3. You know how to estimate what you have to pay for your health care needs in the next year, not including emergencies?				
4. You know where to go for help if you were having trouble affording health insurance outside an employer?				
5. You know what questions to ask so you can choose the best health plan for you?				
6. You would choose the health plan that is best for you?				

Scale 4. Confidence: Using a Health Plan

The next questions ask how confident you feel about **using health insurance**.

How confident are you that…	Not all confident	Slightly confident	Moderately confident	Very confident
1. You know most of the things you need to know about using health insurance?				
2. You know how to find out what is and is not covered before you receive a health care service?				
3. You know how to figure out your share of the cost for care, after the health plan pays their share?				
4. You know what questions to ask your health plan if you have a coverage problem?				

© 2022 Khera N et al. *JAMA Network Open.*

How confident are you that…	Not all confident	Slightly confident	Moderately confident	Very confident
5. You know what to do if your health plan refuses to pay for a service you think should be covered?				

Paez, K.A., Mallery, C.J., Noel, H., Pugliese, C., McSorely, V.E., Lucado, J.L., & Ganachari, D. (October, 2014). Development of the Health Insurance Literacy Measure (HILM): Conceptualizing and Measuring Consumer Ability to Choose and Use Private Health Insurance. *Journal of Health Communication*.

© 2022 Khera N et al. *JAMA Network Open.*

Financial Literacy Scale

1) On a scale of 1 to 7 (1 being very low and 7 being very high), what is your level of financial knowledge?
    a. 1
    b. 2
    c. 3
    d. 4
    e. 5
    f. 6
    g. 7

2) Suppose you had $100 in a savings account and the interest rate was 2% per year. After 5 years, how much do you think you would have in the account if you left the money to grow?
    a. More than $102
    b. Exactly $102
    c. Less than $102
    d. Do not know
    e. Refuse to answer

3) Imagine that the interest rate on your savings account was 1% per year and inflation was 2% per year. After 1 year, how much would you be able to buy with the money in this account?
    a. More than today
    b. Exactly the same
    c. Less than today
    d. Do not know
    e. Refuse to answer

4) If interest rates rise, what will typically happen to bond prices?
    a. They will rise
    b. They will fall
    c. They will stay the same
    d. There is no relationship between bond prices and the interest rates
    e. Do not know
    f. Refuse to answer

5) Please tell me whether this statement is true or false. A 15-year mortgage typically requires higher monthly payments than a 30-year mortgage, but the total interest paid over the life of the loan will be less.
    a. True
    b. False

© 2022 Khera N et al. *JAMA Network Open.*

c. Do not know
d. Refuse to answer

6) Please tell me whether this statement is true or false. Buying a single company's stock usually provides a safer return than a stock mutual fund.

a. True
b. False
c. Do not know
d. Refuse to answer

© 2022 Khera N et al. *JAMA Network Open.*
